# Screening for EGFR Mutations in Patients with Head and Neck Cancer Treated with Gefitinib on a Compassionate-Use Program: A Hellenic Cooperative Oncology Group Study

**DOI:** 10.1155/2010/709678

**Published:** 2011-01-03

**Authors:** Samuel Murray, Mattheos Bobos, Nikolaos Angouridakis, Angelos Nikolaou, Helena Linardou, Evangelia Razis, George Fountzilas

**Affiliations:** ^1^GeneKOR S.A. Glyka Nera, 15354, Athens, Greece; ^2^BioMarker Solutions, 23 Barnsbury Square, London N1 1JP, UK; ^3^Laboratory of Molecular Oncology, Hellenic Foundation of Cancer Research, School of Medicine, Aristotle University of Thessaloniki, 54006, Thessaloniki, Greece; ^4^ENT Department, “AHEPA” Hospital, School of Medicine, Aristotle University of Thessaloniki, 54006, Thessaloniki, Greece; ^5^1st Department of Oncology, “Metropolitan” Hospital, N. Faliro, 18547, Athens, Greece; ^6^2nd Department of Oncology, “Hygeia” Hospital, 15123, Athens, Greece; ^7^Department of Medical Oncology, “Papageorgiou” Hospital, School of Medicine, Aristotle University of Thessaloniki, 54006, Thessaloniki, Greece

## Abstract

*Background and Aim*. EGFR is commonly expressed in cancers of the head and neck (H and N), and anti-EGFR agents have demonstrated improvements in outcomes (TTP and OS). The aim of this study was to determine EGFR gene status in H and N cancer patients treated with gefitinib and to correlate mutational status with clinico-pathological data and response. *Patients and Methods*. Patients with histologically confirmed H and N cancer having failed prior treatment for advanced disease entered this compassionate-use-program. Nineteen patients received gefitinib. EGFR expression was assessed by IHC, gene copy number by FISH, and mutation analysis was conducted for *EGFR* (18-21), *KRAS*, *BRAF* (V600E), and *HER*-2 exon 20. An additional TKI naive cohort of 73 patients was also screened. *Results*. Mutations were detected in 6/19 patients (3× *EGFR*, 1× *KRAS*, and 2× *HER2*-exon 20). There were no significant differences in TTP or OS for patients with somatic *EGFR* mutations. No *BRAF* mutations were detected. *Conclusions*. The incidence of *EGFR* mutations in H and N cancer in this study was 5.3%. No statistically relevant correlations between mutation or gene gain and response or survival were observed. Due to the limited number of patients and low incidence of genetic aberrations in the genes analyzed, additional studies are warranted.

## 1. Introduction

Cancer of the head and neck (H and N) is the fifth most common cancer in the United States, and despite significant progress in therapeutic modalities, almost half of patients with this diagnosis will relapse with local or distant disease, indicating the need for novel therapeutic interventions [1, 2]. Treatment of advanced disease usually involves combinations of chemotherapeutic agents, such as cisplatin, with radiation, while new agents are being studied in platinum-refractory metastatic disease.

The epidermal growth factor receptor (EGFR) along with its ligands epidermal growth factor (EGF) and transforming growth factor alpha (TGF-*α*) are important in many aspects of cell survival, differentiation, proliferation, and invasion [3–5]. EGFR is almost universally expressed in H and N cancers, and high levels of expression have been correlated with poor outcome [6, 7]. Studies have shown that blockade of EGFR signaling using antibody-based approaches (cetuximab) can offer improved outcomes. Recent randomized studies have demonstrated that the combination of radiation with the anti-EGFR monoclonal antibody cetuximab results in improvements of response and overall survival over radiation alone in patients with locally advanced H and N cancer [8]. Combinations of EGFR inhibition with chemotherapy have also been studied with promising results in the metastatic and recurrent settings [9, 10]. Although other EGFR inhibitors, such as small tyrosine kinase inhibitors (TKIs; gefitinib and erlotinib) are available for the treatment of other tumor types; only modest response rates of up to 11% as monotherapy have been obtained in H and N cancers [11, 12]. Considering that the oral TKI erlotinib is licensed for second line NSCLC, and the TKI gefitinib has recently been approved for first line advanced NSCLC carrying somatic *EGFR* mutations, a new level of clinical interpretation may be necessary in this apparently EGFR sensitive disease.

Gefitinib is an orally active and selective inhibitor of the EGFR tyrosine kinase, which has principally been studied in NSCLC. Clinical responses to gefitinib differ among NSCLC patients, and several studies have aimed at identifying prognostic and/or predictive markers of response to these agents. Clinical studies indicated that EGFR TKIs were more effective in women, of Asian origin, individuals with adenocarcinoma, and never smokers [13, 14]. The impact of EGFR expression levels on drug sensitivity is still an open issue, since preclinical and clinical data show no obvious correlation between EGFR immunohistochemical expression and response although no thorough analysis has been performed. *EGFR* gene copy number on the other hand has been shown to be associated with improved response rates and survival outcomes to TKI treatment compared to WT patients, albeit in NSCLC [15, 16]. However, the defining molecular event appears to be the presence of activating sensitizing mutations in *EGFR* [17–19]. These are virtually exclusive to NSCLC but have been reported in numerous other cancers [20]. Patients harboring such mutations have a much higher response rate that is translated into improved survival times compared to WT patients treated with EGFR TKIs, at least in NSCLC [20, 21]. The molecular signatures of NSCLC have also highlighted that the presence of somatic mutations in *KRAS*, occurring mutually exclusively to *EGFR* mutations, earmarks tumors that are essentially resistant to TKIs [22]. Similar molecular events are being unearthed in colorectal cancers with respect to treatment outcomes with the anti-EGFR monoclonal antibodies cetuximab and panitumumab. Here, mutations in key signaling molecules *KRAS* [22], *BRAF* [23], *PIK3CA* [24], and loss of expression of PTEN [25] have been correlated with a lack of response [26].

Many recent studies have consolidated our understanding on the functional blockade of EGFR with various agents. Investigations by numerous groups have now broadened the scope of TKIs by the discovery of similar somatic mutations in other cancer types; however, their correlation with response to TKI treatment is as yet not conclusive. These insights have raised questions as to the effect and incidence of such mechanisms and also as to their prognostic significance.

Although the response rates of H and N cancer to gefitinib are similar to those seen in NSCLC, as yet there appear to be no clinicopathological predictors so far identified for the responsive cases. From limited literature studies in H and N cancer, there are suggestions that *EGFR* mutations similar to those in NSCLC exist, while there are other reports that have failed to detect mutations [27–30]. However, recently there have been reports of the presence of *EGFR* somatic mutations in H and N cancers, albeit at a low incidence (1–14%) [31–37]. Unlike NSCLC where there are multiple studies investigating the predictive nature of gene copy number analysis to TKIs, the data in H and N cancer remains scant and inconclusive [27, 38, 39].

Based on the above, we hypothesized that if somatic mutations and gene copy gain of *EGFR* occur in H and N cancer, then treatment with a TKI such as gefitinib could be a potentially beneficial treatment option for many of these patients. The objective of this study was to determine whether the molecular mechanisms seen in NSCLC regarding *EGFR* mutations and gene copy number and correlation with TKI response extend to H and N cancer. Furthermore, we extended this analysis to examine the incidence of additional molecular events that have been proposed as candidate biomarkers for response to anti-EGFR agents in NSCLC and colorectal cancer.

## 2. Materials and Methods

### 2.1. Patients

Patients with histologically confirmed H and N cancer, who had failed prior treatment for advanced or metastatic disease and were not amenable to further chemotherapy or chemotherapy-naïve patients due to contraindication, were eligible for the study. Eligibility criteria included an ECOG PS of 0–2, estimated life expectancy of at least 3 months, adequate bone marrow, and hepatic and renal function, indicated by an absolute neutrophil count of ≥1,500/*μ*l, platelets ≥75,000/*μ*l, total bilirubin ≤2× the upper limit of normal; serum AST or ALT levels ≤3× the upper limit of normal; serum creatinine ≤2 mg/dl; serum albumin ≥2.5 g/dl. Previous chemotherapy and/or radiotherapy were allowed. All histological subtypes were allowed. Patients with a history of serious cardiac disease, other serious medical illness, or inability to comply with the treatment plan and followup visits were excluded from the study. 

An additional cohort of 37 patients, previously reported [40], were also included in this analysis for comparative purposes. 

A third cohort of anti-EGFR naive patients was also included to aid in the study of the prognostic significance of the biomarkers analysed. These patients were randomly selected from the School of Medicine, University of Thessaloniki, with the only entry criteria being adequate biological material access and patient's informed consent for biomarker analysis.

All patients signed informed consent as a requirement for study inclusion. They similarly signed consent for the use of biological materials for research purposes. The study was conducted according to the Declaration of Helsinki and the guidelines for Good Clinical Practice. The Local Ethics Committees approved the study and the collection of biological material.

### 2.2. TKI Treatment

Patients received gefitinib at a dose of 250 mg per day orally. Gefitinib was supplied free of charge by AstraZeneca as part of a compassionate use program. Treatment was administered daily with a treatment cycle constituting 28 days. Treatment was discontinued for up to 7 days for grade 3 or 4 toxicity, until resolution of toxicity to ≤1. For non-resolving toxicities of more than 15 days, patients were taken off study. Treatment was continued until disease progression, serious adverse toxicity, at the direction of the treating physician, or following patient withdrawal. Patients were eligible for response evaluation after completion of at least 2 months of treatment. All patients have been routinely followedup at 6 monthly intervals from their last treatment (data on file, HeCOG Data Office). Complete clinical data including smoking history, clinical stage, pathological diagnosis, and response data for all patients was available.

### 2.3. Determination of EGFR Expression

Paraffin blocks of tumor were collected retrospectively, and peripheral blood samples were collected during treatment or followup. Immunohistochemical detection of EGFR was performed as previously described [41] to semiquantify EGFR expression levels. Tumor tissue sections showing 2^+^ or 3^+^ were considered as positive.

Assessment of *EGFR* gene copy number was conducted by FISH and scored as previously described [40, 41].

### 2.4. Mutation Analyses

Genomic DNA was extracted from paraffin embedded tumors as previously described [42]. All paraffin blocks were examined on H and E for histological verification according to WHO [43], tumors consisting of >75% tumor cell content (% TCC) were considered as eligible for DNA extraction and sequence analysis. For those biopsies where the % TCC was inadequate, macrodissection on 5 *μ*m sections was performed to increase the content to >75%.

Mutational analysis for all genes was conducted as previously described [40].

Additional genes for which analysis was conducted included* KRAS* mutation analysis of codons 12 and 13. PCR was performed using the same conditions as for *EGFR,* using *KRAS*-specific primers amplifying Exon 2 as previously described [40]. *BRAF* exons 14 and 15 were analyzed as previously described [40], and the 3′ and 5′ intron-exon splice sites of *MET* exon 14 were also screened. The primer sequences for all reactions are available upon request.

All studied exons were confirmed as previously described above for *EGFR* [40]. All PCR products were purified by solid-phase reversible immobilization chemistry followed by bidirectional dye-terminator fluorescent sequencing. Sequences were analyzed by BLAST and chromatograms by manual review and compared to the following representative gene accession numbers: *EGFR,* NM_005228, and/or the *EGFR* gene sequence accession number: AF288738; *KRAS,* gi: 14277199; *HER2* exon 20, gi: 23462913; *MET*, gi: 212720875 (http://www.ncbi.nlm.nci/).

The *EGFR* exon 21 mutation L858R which represents approximately 40% of all reported mutations in NSCLC [44] was also analyzed by PCR-RFLP based on the presence of a new Sau96I restriction site created by the mutation [40]. *KRAS* mutations of codons 12 and 13 were also analyzed by PCR-RFLP based on modified versions of the protocols of Boldrini et al. [45] and Kislitsin et al. [46]. *MET* exon 14 5′ and 3′ intron-exon deletions first reported in NSCLC [47] were also analyzed by a mutant allele-specific PCR method, which only amplifies in the presence of the given deletions, while *BRAF *V600E was also analyzed by PCR-RFLP based on a modified version of Salvesen et al. [48].

### 2.5. Statistical Analysis

Endpoints included TTP (time to progression) and survival in association with the candidate biomarkers. Survival was defined as the time from first day of treatment until death from any cause. TTP was computed as the time from initiation of treatment until recurrence of tumor or death from any cause. The Fisher's exact test was used for comparing groups of categorical data, while for continuous data the Mann-Whitney test. *P*-values of at least .05 were considered statistically significant. Kaplan-Meier curves and log-rank test were used for comparing time to event distributions. All analyses were performed using SPSS version 18, in the HeCOG data office.

## 3. Results

### 3.1. Clinicopathological Charateristics of Whole Cohort

Characteristics of the 73 TKI naive patients are listed in Table 1. A subcohort of these patients received the anti-EGFR agent cetuximab, and details of these patients have been previously presented [49]. In the TKI treatment group, a total of 19 patients were enrolled into a compassionate-use program between 7/2002 and 11/2005. Gefitinib-treated patients were predominantly white males, median age 66 years, with median PS 1. The majority of patients had a strong smoking history (1 unknown), and the majority had received prior chemotherapy (79%). Four patients had never received chemotherapy (i.e., treatment naïve) entering the compassionate use study due to contraindication to standard chemotherapy. Since this study was not designed to determine the response rate, TTP, or survival to treatment with gefitinib, it was not deemed necessary to analyze untreated patients in this investigational/translational study.

The second cohort of untreated (TKI naive) patients had similar patient and tumor characteristics.

### 3.2. TKI-Treated Cohort

#### 3.2.1. Treatment Characteristics

Patients received a total of 98 cycles of treatment (median 4 cycles, range 1–16 cycles). At the time of analysis, all patients had died. Reasons for treatment discontinuation included progression (5/19), death (1/19), and patient refusal (2/19).

#### 3.2.2. Response, TTP, and Survival

A total of 4 (21.1%) of patients achieved an overall objective response (CR + PR) and further 6 (31.6%) achieved disease stabilization. Interestingly only one of the responding patients had gene copy number gain; the 3 patients with *EGFR* mutations demonstrated disease stabilization. There were 19 disease progressions for a median TTP of 3.6 months (95% CI: 1.5–5.6) and a total of 19 deaths for a median survival of 6.5 months (95% CI: 2.4–10.6).

#### 3.2.3. Somatic Mutation Analysis

**Figure 1:**
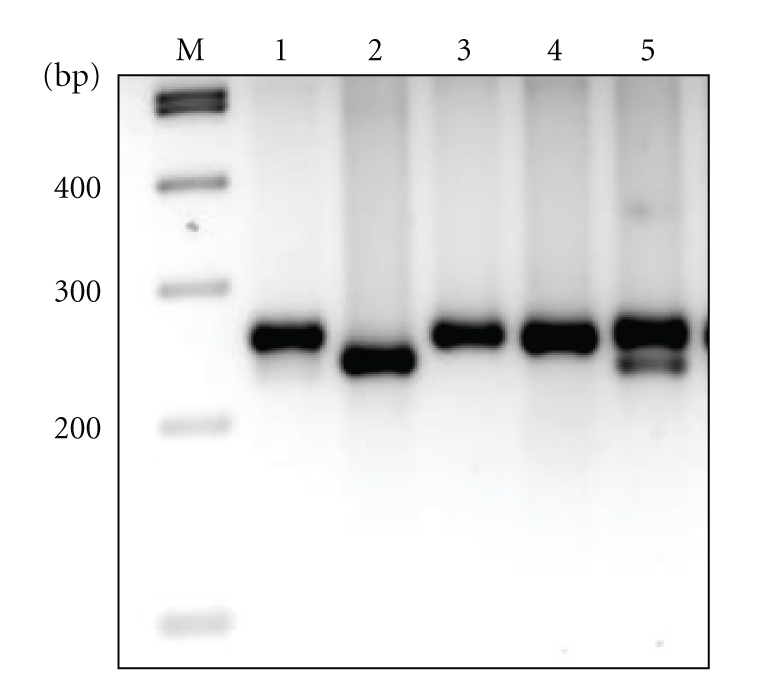
EGFR Exon 19 deletions. M: molecular weight markers; 1, 3, 4: WT Exon 19 EGFR; 2: delL747-P753insS Exon 19 and EGFR gene amplification; 5: delL747-P753insS Exon 19 EGFR.

**Figure 2:**
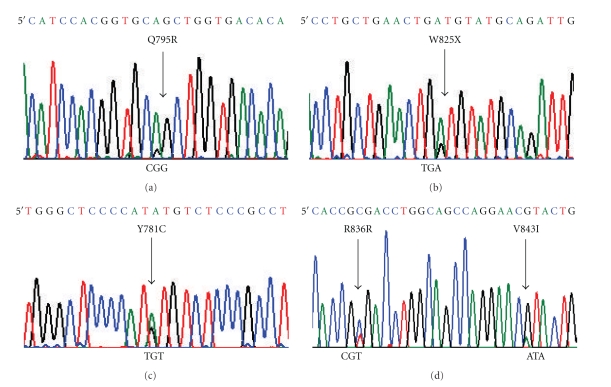
Representative chromatograms of somatic mutations (sense 5′) of (a): HER2 Exon 20 Q795R; (b): HER2 Exon 20 W825X (Stop); (c): HER2 Exon 20 Y781C; (d) EGFR Exon 21, R836R, and V843I.

We performed mutational analysis of exons 18, 19, 20, and 21 of biopsy material from 19 patients with H and N cancer treated with Gefitinib. In this cohort, a total of 4 different somatic mutations located within the exons of the TK domain of *EGFR* were observed in 3 patients. Mutational status of all mutations was confirmed using germline DNA extracted from peripheral blood or from macrodissected normal tissue available from the paraffin embedded biopsy. Two patients harbored delL747-P753insS (one with associated gene amplification), Figure 1, and another patient harbored V843I with a secondary silent R846R somatic mutation (also in the presence of *EGFR* gene amplification), Table 4 and Figure 2.

No patient was found to harbor the common exon 21 mutation L858R either on sequencing or by RFLP. *KRAS* mutations were observed in 1 patient, occurring at codon 12. *EGFR* and *KRAS* mutations were mutually exclusive. No patient was found to harbor a *BRAF* V600E point mutation, nor was there any other mutations observed in exon 15 of the *BRAF* gene. Similarly no analyzed patient was found to harbor an inton-exon 14 deletion of the *MET* gene. Two patients were found to have somatic mutations within exon 20 of *HER-2*, one harboring two independent point mutations, Table 5, Figure 2.

Somatic mutations were detected in 6/19 patients (3×*EGFR*, 1×*KRAS*, and 2×*HER2*-exon 20). The incidence of *EGFR* mutations in this population was 15.8% (3/19). Patients with somatic *EGFR* Exon19 deletions and point mutations in Exon21 had a longer TTP and survival compared to patients without mutations (not statistically significant), as shown in Table 4. For patients without mutations, TTP was 2.2 m versus 7.7 m for patients with mutations (Log Rank *P* = .145), and survival was 4.6 m for patients without mutations versus 11.6 m for mutation carriers (Log Rank *P* = .125). Similarly no statistical significant difference was found with respect to any other biomarker analyzed in this study.

Immunohistochemical analysis for *EGFR* expression identified positive tumors in 76.5% (13/17) of cases. Three patients had gene amplification of *EGFR* (two with concomitant somatic mutations in *EGFR*), and one additional patient had gene copy number gain (aneuploidy), Table 5. Although *EGFR* copy gain was not associated with a prolonged survival (survival was 11.64 m), when we combined patients with a somatic *EGFR* mutation and/or gene copy number gain, they performed better (but not statistical significant) compared to the wild-type (WT) group, 4.6 to 11.6 months, for survival; 2.2 to 7.7 months for TTP (WT versus mutation/gene gain, resp.).

### 3.3. Naive Cohort

The TKI naive cohort consisted of additional 73 patients, Tables 1 and 2. Of these patients, 37 have previously been analyzed for *EGFR* and *KRAS *mutational status [49]. These patients were included to obtain more accurate estimation of the frequency of gene alterations present in H and N cancers. The overall incidence per aberration analyzed is indicated in Table 3.

## 4. Discussion

We have analyzed three differently selected populations of H and N cancer in order to further understand the incidence and potential predictive ability of a subset of specific anti-EGFR candidate biomarkers. Although the representative population of TKI-treated patients was small, the incidence of both *EGFR* somatic mutations and gene gain/amplification was relatively high (3 and 4, resp., (2 coexistent)) leading us to incorporate additional 73 patients to more closely reflect their respective incidence in a more balanced population.

With the inclusion of 92 patients, the overall incidence of *EGFR* mutations indicates that at 4% (4/92) they are rare among cases of H and N cancer within the Greek population. A number of other authors have reported on the presence of *EGFR* kinase domain mutations in H and N cancers. These have ranged from not being detected from a total of 221 patients [27–30], through to between 1 and 14% in other studies (*n* = 15/425) [31–37]. These differences suggest that such mutations are a rare event, but that ethnicity or other cofactors may play a role. Indeed, differences in incidence have been well characterized with respect to *EGFR* mutations in NSCLC, wherein the incidence is twice that in Asians compared to Whites [20]. Bearing in mind that the TKI gefitinib has recently gained license approval for the treatment of first line NSCLC for patients harboring *EGFR* mutations [50], the search for similar biomarkers of response to anti-EGFR agents has become a priority in virtually all cancer types.

Head and neck cancers are not without their fair share of responses to anti-EGFR therapies. There are a number of studies that have reported similar response rates of H and N cancer to TKIs as to the response rates observed with single agent TKIs in unselected NSCLC [11, 12]. Further to this is the clinical utilization of anti-EGFR monoclonal antibodies including cetuximab in the treatment of H and N cancers [8]. Given such utility and with the knowledge that none of the biomarkers analyzed herein were found to correlate with outcomes in the FLEX [51] study, each of these biomarkers will need to be more thoroughly investigated in H and N cancer. We also investigated the incidence of *EGFR* gene copy gain finding 5.3% (1/19) of such patients. This matches previous data indicating that between 8 and 30% of esophageal cancers have *EGFR* gene gain [52, 53]. As with *EGFR* somatic mutations, there is currently little evidence to speculate on the overall predictive nature of these aberrations in H and N cancers receiving anti-EGFR-based agents. There are of course some limitations to interpretations being derived from this study, the major of which is the small sample size respective to the TKI-treated population. Although there were differences between the *EGFR* mutation positive and negative groups, their numbers were small and any difference could simply be attributable to chance. The relatively low incidences of such mutations in H and N cancer, as well as mutations in *KRAS*, *BRAF,* and *HER2*, indicate the necessity for analysis of much larger patient populations together with investigation of alternative molecular pathways and mechanisms to identify predictive or prognostic markers.

In the continued search for additional biomarkers that may be predictive of response to anti-EGFR agents, we and others [12] have extrapolated data from breast cancer [54], colorectal cancer [22, 26], and NSCLC [20] in order to address additional candidate genes/biomarkers to receptor tyrosine kinases in general. As indicated in our cohorts, no somatic mutations were identified in *BRAF* or *MET*. Only one other report has analyzed *HER2* mutations, suggesting that they may be a potential biomarker of EGFR-TKI sensitivity [12]. Although our investigation did not expand into the other 2 cohorts, the Erbitux-treated cohort previously investigated [49] was also analyzed for the presence of PTEN loss. Both PTEN and *PIK3CA* remain potential candidate biomarkers of response to anti-EGFR agents. Further analyses should incorporate analysis of these two molecular events, as preliminary data suggests that *PIK3CA* mutations may be as common as 20% [55] and PTEN expression may be of predictive/prognostic significance [56]. The limited data that currently exists not only to anti-EGFR agents but also to chemotherapy in general in H and N cancers beckons further concerted efforts into additional molecular taxinometry in order to start to substratify patient populations for treatment individualization.

In conclusion, from a relatively small cohort of TKI-treated H and N cancers, there is little evidence of any single biomarker or biomarker algorithm from the genes investigated as capable of subclassifying H and N cancer into two distinct responsive subgroups. Although differences in survival endpoints did not reach statistical significance between these groups, there are many contributing factors to small retrospective analyses, such as inappropriate patient selection and/or reporter bias compounding the analysis. Considering that there are (to date) no effective biomarkers in H and N cancer, additional studies are welcome in order to identify and further clarify if any underlying mechanisms of response or resistance to anti-EGFR agents exist in H and N cancer.

##  Conflict of Interests

*Consultant or Advisory Role*. Dr. S. Murray, Merck KGaA, Darmstadt, Germany. Merck distributes the MoAb Cetuximab (Erbitux); AstraZeneca, Maccelsfield, United Kingdom. AstraZeneca are proprietors of gefitinib (Iressa); Amgen Thousand Oaks, Ca, USA. Amgen distributes the MoAb Panitumumab (Vectibix). Dr. S. Murray is an employee of Biomarker Solutions; in this role, he has no conflict of interests with proprietary agents mentioned in this paper. No other author has a conflict of interests.

##  Funding Source

This study was solely funded by HeCOG.

## Figures and Tables

**Table 1:** Patient and tumor characteristics by group.

	TKI treated		Naive	
	Number	%	Number	%
Total	19	100	73	100
Gender				
Male	19	100	60	82.2
Female			13	17.8
Age (years)				
Median	66		60
Range	38–75		36–82
Histological type				
SCC	18	94.7	69	94.5
Other	1	5.3	2	2.7
Unknown			2	2.7
Smoking status				
Yes	18	94.7	64	87.7
No			8	11.0
Unknown	1	5.3	1	1.3
Alcohol				
Yes	11	57.9	56	76.7
No	7	36.8	15	20.5
Unknown	1	5.3	2	2.7

**Table 2:** Molecular characteristics per group.

	TKI treated		Naive	
	(*N* = 19)		(*N* = 73)	
	*N*	%	*N*	%
RAS				
MUT	1	5.3		
WT	18	94.7	73	100.0
EGFR				
MUT	3	15.8	1	1.4
WT	16	84.2	56	76.7
Unknown			16	21.9
MET				
WT	19	100.0	36	49.3
Unknown			37	50.7
BRAF				
WT	19	100.0		
Unknown			73	100.0
HER2 exon 20				
MUT	2	10.5		
WT	17	89.5		
Unknown			73	100.0
EGFR (IHC)				
0	4	21.1		
1			2	2.7
2	3	15.8	8	11.0
3	10	52.6	22	30.1
NE	2	10.5	3	4.1
Unknown			38	52.1
EGFR (FISH)				
Amplified	3	15.8	1	1.4
Nonamplified	13	68.4	32	43.8
NE	3	15.8		
Unknown			40	54.8

**Table 3:** Survival according to molecular markers for TKI-treated cohort.

	Survival	Log rank *P*	TTP	Log rank *P*
EGFR				
MUT	11.7	.131	8.5	.106
WT	5.1		2.7	
HER2				
MUT	2.7	.340	1.2	.100
WT	6.5		4.1	
EGFR (IHC)				
Negative	8.9	.700	1.2	.641
Positive	4.6		3.6	
EGFR (FISH)				
Negative	4.6	.271	3.1	.185
Positive	11.7		8.5	
EGFR combination: (Mutation and/or Gene gain)				
Negative	4.6	.125	2.2	.145
Positive	11.6		7.7	

**Table 4:** Response correlations. Patients, tumor characteristics and mutations by response for TKI treated cohort.

	CR or PR (*N* = 4)		SD or PD (*N* = 13)		
	*N*	%	*N*	%	*P*
Alcohol					.999
No	2	50.0	5	41.7	
Yes	2	50.0	7	58.3	
RAS					.235
MUT	1	25.0			
WT	3	75.0	13	100.0	
EGFR					.541
MUT			3	23.1	
WT	4	100.0	10	76.9	
HER2					.999
MUT			1	7.7	
WT	4	100.0	12	92.3	
EGFR (IHC)					.516
Negative			4	33.3	
Positive	3	100.0	8	66.7	
EGFR (FISH)					.999
Negative	3	100.0	8	72.7	
Positive			3	27.3	

**Table 5:** Characteristics of patients receiving Gefitinib.

						Somatic Mutational Spectrum					EGFR	
No	Age	Previous Chemo-therapy	Response	TTP (months)	Survival (months)	RAS 12/13	EGFR Exons 18–21	BRAF exon 15	MET 5′–3′ exon 14 intro-exon boundaries	Her2 Exon20	FISH	IHC
1	38	Y	PD	1.25	8.85	WT	WT	WT	WT	Y781C	ND	0
2	50	Y	PR	2.20	5.70	WT	WT	WT	WT	WT	ND	ND
3	75	Y	SD	5.31	6.46	WT	WT	WT	WT	WT	WT	0
4	59	Y	NE	2.66	2.66	WT	WT	WT	WT	Q795R	WT	3
										W825X		
5	63	Y	SD	12.13	12.13	WT	delL747-P753insS	WT	WT	WT	Amp	ND
6	66	Y	PD	2.07	2.43	WT	WT	WT	WT	WT	WT	3
7	63	Y	SD	7.74	9.54	WT	delL747-P753insS	WT	WT	WT	WT	0
8	72	Y	SD	8.46	11.70	WT	R836R	WT	WT	WT	Amp	3
							V843I					
9	62	Y	SD	7.84	8.03	WT	WT	WT	WT	WT	ND	3
10	72	Y	PR	3.11	3.11	WT	WT	WT	WT	WT	WT	3
11	75	Y	PD	5.15	5.15	WT	WT	WT	WT	WT	Amp	2
12	66	N	PR	4.10	11.64	WT	WT	WT	WT	WT	Gain	2
13	74	N	PD	3.57	9.15	WT	WT	WT	WT	WT	WT	3
14	61	N	PD	1.64	3.74	WT	WT	WT	WT	WT	WT	3
15	70	N	CR	15.05	17.57	G12D	WT	WT	WT	WT	WT	3
16	69	Y	PD	0.82	10.75	WT	WT	WT	WT	WT	WT	0
17	63	Y	PD	0.75	2.89	WT	WT	WT	WT	WT	WT	3
18	72	Y	NE	4.59	4.59	WT	WT	WT	WT	WT	WT	3
19	59	Y	SD	1.48	3.80	WT	WT	WT	WT	WT	WT	2

Abbreviations: Y: Yes; N: No; IHC: immunohistochemistry; FISH: fluorescent in situ hybridization; ND: not determined; gain: aneuploidy; CR: complete response; PR: partial response; SD: stable disease; PR: progressive disease; NE: not evaluable.
